# Both Clathrin-Mediated and Membrane Microdomain-Associated Endocytosis Contribute to the Cellular Adaptation to Hyperosmotic Stress in *Arabidopsis*

**DOI:** 10.3390/ijms222212534

**Published:** 2021-11-21

**Authors:** Zheng Wu, Chengyu Fan, Yi Man, Yue Zhang, Ruili Li, Xiaojuan Li, Yanping Jing

**Affiliations:** 1 National Engineering Laboratory for Tree Breeding, College of Biological Sciences and Technology, Beijing Forestry University, No. 35 Qinghua East Road, Beijing 100083, China; wuzheng0827@bjfu.edu.cn (Z.W.); manyi@bjfu.edu.cn (Y.M.); yue_zhang@bjfu.edu.cn (Y.Z.); liruili@bjfu.edu.cn (R.L.); lixj@bjfu.edu.cn (X.L.); 2 Key Laboratory of Genetics and Breeding in Forest Trees and Ornamental Plants, Ministry of Education, Beijing Forestry University, No. 35 Qinghua East Road, Beijing 100083, China; 3 Molecular Imaging Core Facility, School of Life Science and Technology, ShanghaiTech University, 393 Middle Huaxia Road, Shanghai 201210, China; fanchy@shanghaitech.edu.cn; 4 The Tree and Ornamental Plant Breeding and Biotechnology Laboratory of National Forestry and Grassland Administration, Beijing Forestry University, No. 35 Qinghua East Road, Beijing 100083, China

**Keywords:** endocytosis, clathrin, microdomain, hyperosmotic stress, *Arabidopsis thaliana*

## Abstract

As sessile organisms, plants must directly deal with an often complex and adverse environment in which hyperosmotic stress is one of the most serious abiotic factors, challenging cellular physiology and integrity. The plasma membrane (PM) is the hydrophobic barrier between the inside and outside environments of cells and is considered a central compartment in cellular adaptation to diverse stress conditions through dynamic PM remodeling. Endocytosis is a powerful method for rapid remodeling of the PM. In animal cells, different endocytic pathways are activated in response to osmotic stress, while only a few reports are related to the endocytosis response pathway and involve a mechanism in plant cells upon hyperosmotic stress. In this study, using different endocytosis inhibitors, the microdomain-specific dye di-4-ANEPPDHQ, variable-angle total internal reflection fluorescence microscopy (VA-TIRFM), and confocal microscopy, we discovered that internalized Clathrin Light Chain-Green Fluorescent Protein (CLC-GFP) increased under hyperosmotic conditions, accompanied by decreased fluorescence intensity of CLC-GFP at the PM. CLC-GFP tended to have higher diffusion coefficients and a fraction of CLC-GFP molecules underwent slower diffusion upon hyperosmotic stress. Meanwhile, an increased motion range of CLC-GFP was found under hyperosmotic treatment compared with the control. In addition, the order of the PM decreased, but the order of the endosome increased when cells were in hyperosmotic conditions. Hence, our results demonstrated that clathrin-mediated endocytosis and membrane microdomain-associated endocytosis both participate in the adaptation to hyperosmotic stress. These findings will help to further understand the role and the regulatory mechanism involved in plant endocytosis in helping plants adapt to osmotic stress.

## 1. Introduction

Cells must deal with stochastic and challenging changes in their environment. A prominent example is alterations in osmolarity. Deviations from homeostatic conditions are often unfavorable to cellular life and evoke stress responses, thus enabling cell adaptation and survival under adverse environments [1,2,3]. The plasma membrane (PM) is considered a central compartment in cellular adaptation to diverse stress conditions since it is the barrier between the inside and outside environments of cells [4]. Therefore, dynamic PM remodeling is a crucial process in cellular adaptation to various stress conditions.

Endocytosis provides a major route of entry for membrane proteins, lipids, and extracellular molecules into the cell. Endocytosis is a powerful method for rapid remodeling of the plasma membrane [5]. Furthermore, endocytosis plays an important role in nutrient uptake, signal transduction, stress response, and plant–microbe interactions [6,7]. Similar to mammalian cells, clathrin-mediated endocytosis (CME) is the major endocytic pathway in plants. During the process of CME, clathrin-coated vesicles (CCVs) are formed by the coordinated assembly of clathrin triskelia built from three clathrin heavy chain (CHC) and three clathrin light chain (CLC) subunits onto the PM [8,9]. In addition to CME, plant cells have additional endocytic pathways in the clathrin-independent route. Membrane rafts are rich in sphingolipids and cholesterol; they are liquid-ordered membrane microdomains with a unique protein such as flotillin1 (Flot1). Flot1-assisted membrane microdomain-associated endocytosis discovered in *Arabidopsis thaliana* has been found to be involved in the regulation of signal transduction via endocytosis [10,11,12,13].

Hyperosmotic stress, one of the most serious abiotic factors that have adverse effects on plant growth and development, can affect the uptake of water and nutrients by plants from the soil and lead to premature senescence and even death of plants [14,15]. Many environmental challenges, such as drought and salinity, can lead to hyperosmotic stresses [16,17,18,19]. Hyperosmotic stress is likely perceived by the cell as turgor loss or changes in PM tension, which are thought to influence endocytic pathways [20,21,22]. Studies from mammalian cells have indicated that elevated tension on the membrane hinders CME, acting as a reversible regulator of CME [23,24]. In filamentous fungi, moderate hyperosmotic stress had no effect on the dynamics of early endocytosis [25]. Transient inhibition of CME and enhanced caveolar endocytosis were reported in animal cells under modest hyperosmotic stress, suggesting that the effect of hyperosmotic stress on endocytosis may be related to species, and different endocytosis pathways may respond to hyperosmotic stress in various ways.

In plants, tension on the plasma membrane was proposed to determine the addition or removal of membranes at the cell surface [21]. Plant cells are reported to respond to hyperosmotic stress by accelerating endocytosis to internalize excess PM [13]. However, only a few reports are related to the endocytosis response pathway and involve a mechanism in plant cells upon hyperosmotic stress. Here, we explained the regulatory mechanism of plant cell adaptation to hyperosmotic stress by studying the involvement of different endocytosis pathways in *Arabidopsis* root epidermal cells.

## 2. Results

### 2.1. Hyperosmotic Stress Promoted the Diffusion and Endocytosis of Clathrin at the Plasma Membrane

**Figure 1:**
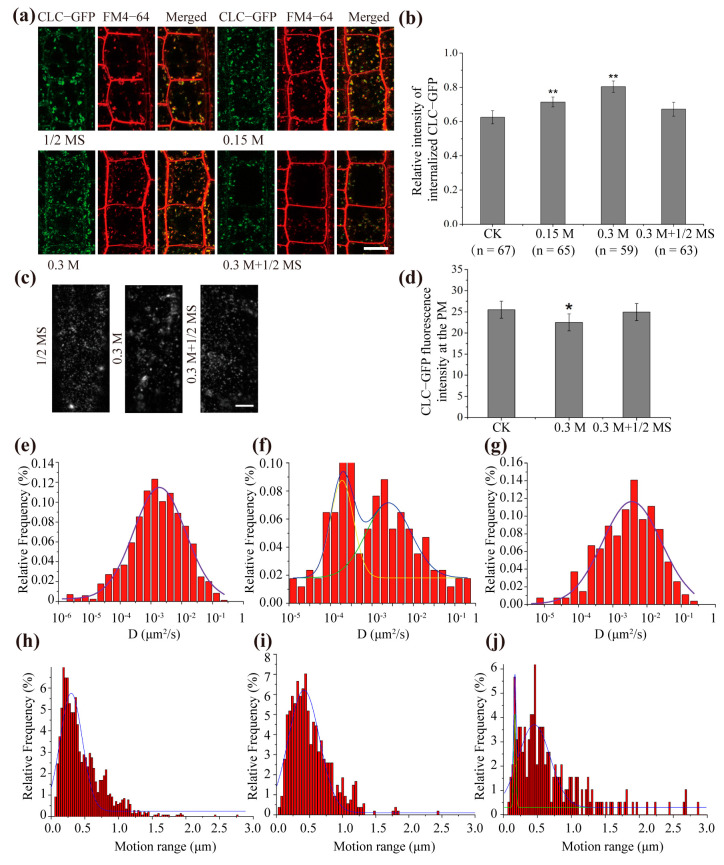
The endocytosis and dynamic behavior of Clathrin Light Chain-Green Fluorescent Protein (CLC-GFP) in response to hyperosmotic stress. (**a**) Images of root epidermal cells of 4-day-old *Arabidopsis thaliana* seedlings expressing CLC-GFP stained with FM4-64 under different conditions: CK (1/2 MS), hyperosmotic stress (0.15 M mannitol, 0.3 M mannitol) and recovery from hyperosmotic stress (0.3 M mannitol + 1/2 MS). Bar = 10 μm. (**b**) The relative fluorescence intensity of internalized CLC-GFP in different treatments. Asterisks mark significant differences (Student’s *t*-test, ** *p* < 0.01); *n* = number of cells analyzed. (**c**) Variable-angle total internal reflection fluorescence microscopy (VA-TIRFM) images of root cells in *Arabidopsis thaliana* in the 1/2 MS-treated control and in cells exposed to 0.3 M mannitol and in cells after recovery from hyperosmotic treatment (0.3 M mannitol + 1/2 MS). Bar = 10 μm. (**d**) Quantification of the fluorescence intensity of CLC-GFP at the plasma membrane (PM) in different treatments. Asterisks mark significant differences (Student’s *t*-test, * *p* < 0.05). (**e**–**g**) Distribution of CLC-GFP diffusion coefficients in seedlings treated with 1/2 MS (**e**), 0.3 M mannitol (**f**) and 0.3 M mannitol + 1/2 MS (**g**). (**h**–**j**) Motion range of CLC-GFP in seedlings treated with 1/2 MS (**h**) and 0.3 M mannitol; (**i**) and seedling recovery after hyperosmotic treatment (0.3 M mannitol + 1/2 MS, j).

CME is the main endocytic pathway in plants. To explore the involvement of CME under hyperosmotic stress, we first followed the internalization and dynamics of clathrin at the PM. Using an artificially constructed Clathrin Light Chain-Green Fluorescent Protein (CLC-GFP) fusion protein, which was previously used and proved to be functional [26,27], and the endocytic tracer dye FM4-64, we found that internalized CLC-GFP colocalized with FM4-64 signals and increased under hyperosmotic stress (Figure 1a). Both internal CLC-GFP intensity and FM4-64 intensity increased with increasing mannitol concentration (Figure 1a), and the relative CLC-GFP intensity increased significantly by 14.5 ± 2.3% and 29.1 ± 3.5% upon 0.15 M and 0.3 M mannitol (Figure 1b), suggesting that hyperosmotic stress enhanced the internalization of clathrin. In addition, the internalization of clathrin was restored when the hyperosmotic conditions were relieved, indicating that the clathrin-mediated endocytosis caused by hyperosmotic stress was reversible (Figure 1a,b). To investigate the movement of CLC-GFP on the PM upon hyperosmotic treatment, we used a variable-angle total internal reflection fluorescence microscopy (VA-TIRFM) to track the protein and found fluorescent spots diffracted by incident light formed on the PM (Figure 1c). The fluorescent dots on the PM were highly dynamic, the spots marked by CLC-GFP tended to aggregate, and the relative fluorescence intensity of CLC-GFP at the PM decreased to 94.1 ± 3.4% of the relative fluorescence intensity of CLC-GFP at the PM of the control under hyperosmotic stress (Figure 1c,d). The change was greatly reversed after hyperosmotic stress was relieved, and the fluorescence intensity recovered to 97.9 ± 1.9% of the control (Figure 1c,d).

We then determined the dynamic properties of CLC-GFP proteins in more detail by calculating the diffusion coefficient and the motion range under hyperosmotic stress. The diffusion coefficient (D) is often defined as the ratio of flux density to the negative of the concentration gradient in direction of diffusion, and it is always used to quantify the movement of molecules [28]. The distribution of diffusion coefficients was plotted on a histogram and fitted using Gaussian functions in which the peaks (Ĝ) were regarded as the characteristic value of the diffusion coefficients. The results showed that Ĝ was 1.95 ± 0.15 × 10^−^^3^ μm^2^/s in cells under control conditions (Figure 1e). Upon hyperosmotic stress treatment, the frequency histogram of the diffusion coefficient showed an obvious bimodal distribution trend, and Ĝ was 2.43 ± 0.09 × 10^−^^3^ μm^2^/s and 3.83 ± 0.12 × 10^−^^4^ μm^2^/s, respectively (Figure 1f). When the hyperosmotic stress was relieved, the diffusion coefficient of CLC-GFP on the PM returned to a unimodal distribution and Ĝ recovered close to the control (Figure 1g). In addition, we calculated the motion ranges and found that the histogram of the motion range under normal conditions showed a unimodal distribution with the peak value of 0.185 ± 0.00947 μm (Figure 1h). Compared with the normal conditions, 0.3 M mannitol treatment caused a significant increase in the motion range (peak value of 0.4703 ± 0.02105 μm) (Figure 1i). After the hyperosmotic stress was recovered, the motion range changed to a bimodal distribution, suggesting the existence of two subpopulations of diffusing CLC-GFP particles. Among which, one subpopulation of the CLC-GFP particles showed less mobility, with a peak value similar to the particles under normal conditions (0.181 ± 0.00588 μm) (Figure 1i), whereas another subpopulation showed a peak value of 0.45418 ± 0.01857 μm (Figure 1j). These results indicated that upon hyperosmotic stress, the motion range of CLC-GFP molecules increased and partially recovered after the hyperosmotic stress was relieved.

### 2.2. Clathrin-Mediated Endocytosis Is Involved in Responding to Hyperosmotic Stress

**Figure 2:**
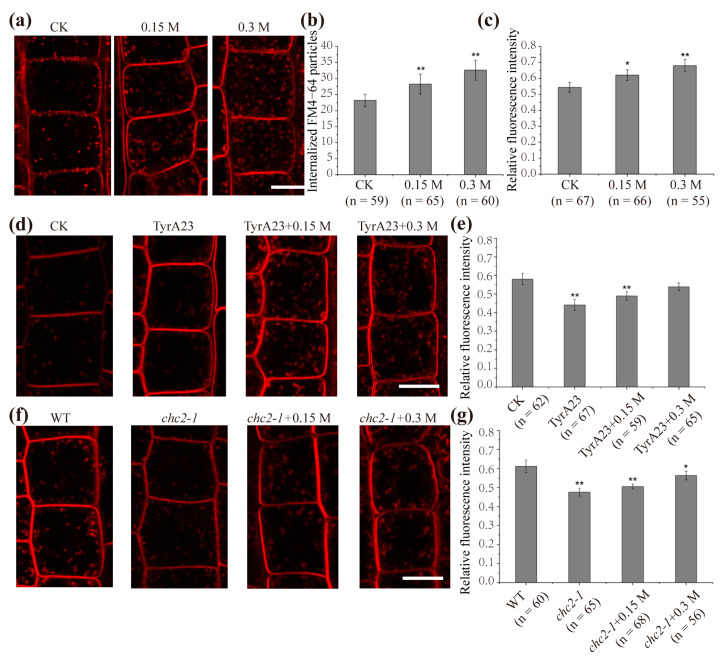
Increased endocytosis in response to hyperosmotic stress is partially dependent on CME. (**a**) FM4-64 uptake in *Arabidopsis* root epidermal cells treated with 1/2 MS, 0.15 M mannitol, and 0.3 M mannitol. (**b**) Quantification of internalized FM4-64 particles. (**c**) Quantification of the intracellular relative FM4-64 fluorescence intensity of (a). (**d**) FM4-64 uptake in *Arabidopsis* root epidermal cells treated with 1/2 MS (CK) and in the presence of 50 μM CME inhibitor under different conditions: TyrA23 (1/2 MS), hyperosmotic stress (0.15 M mannitol, 0.3 M mannitol). (**e**) Quantification of the intracellular relative FM4-64 fluorescence intensity of (**d**). (**f**) FM4-64 uptake in *Arabidopsis* root epidermal cells treated with 1/2 MS, and FM4-64 uptake in *chc 2-1* under different conditions: 1/2 MS, hyperosmotic stress (0.15 M mannitol, 0.3 M mannitol). (**g**) Quantification of the intracellular relative FM4-64 fluorescence intensity of (**f**). Asterisks mark significant differences (Student’s *t*-test, ** *p* < 0.01; * *p* < 0.05); *n* = number of cells analyzed. Bar = 10 μm.

To assess how endocytosis changed in root epidermal cells under hyperosmotic stress, we imaged cells upon hyperosmotic stress (0.15 M and 0.3 M mannitol) with the endocytic tracer dye FM4-64. Compared with CK, we noted a significant increase in FM4-64 signal intensity as hypertonicity increased (Figure 2a). By digitizing the internal quantities of FM4-64 particles, we found 19.6 ± 7.5% and 41.3 ± 6.9% increases under 0.15 M and 0.3 M mannitol treatment, respectively, compared with those under the control condition (Figure 2b). When the relative FM4-64 internal signal intensity was considered, 18.6 ± 7.5% and 40.3 ± 6.9% increases under 0.15 M and 0.3 M mannitol treatment were observed, implying increased endocytosis under hyperosmotic stress (Figure 2c). To explore whether the CME pathway was involved in this process, we used the CME pathway inhibitor Tyrphostin A23 (TyrA23). Compared with the control, we found a substantial reduction in endocytosis (relative FM4-64 internal signal intensity decreased from 0.58 ± 0.03 to 0.43 ± 0.02) when the CME inhibitor TyrA23 was applied (Figure 2d,e). Moreover, we treated root epidermal cells with both TyrA23 and different concentrations of mannitol. The root epidermal cells still showed a reduction compared with the control but resulted in a 9.3 ± 2.7% (0.15 M mannitol) and 23 ± 2.4% (0.3 M mannitol) increase, respectively, compared with single TyrA23 treatment (Figure 2e). We further tested the changes in endocytosis in the clathrin loss-of-function mutant *chc2-1* upon hyperosmotic stress. Similar to the TyrA23 treatment, *chc2-1* mutant showed a marked decrease in endocytosis (from 0.61 ± 0.03 to 0.49 ± 0.01) compared with control wild-type seedlings. After different hyperosmotic treatments, the intracellular relative fluorescence intensity of FM4-64 increased by 6.4 ± 1.2% (0.15 M mannitol) and 19.1 ± 4.6% (0.3 M mannitol), respectively (Figure 2f,g). In summary, the average endocytosis increase was approximately 18.6% (0.15 M mannitol) and 40.3% (0.3 M mannitol) without TyrA23 treatment, and the average endocytosis increase was slowed down to 9.3% (0.15 M mannitol) and 23.0% (0.3 M mannitol), respectively, when CME was inhibited. In *chc2-1* mutants, the overall endocytosis increase slowed to 6.4% and 19.1%, respectively. Hence, CME is involved in responding to hyperosmotic stress and is enhanced during this process. Some endocytosis was still observed, even after blocking the CME pathway with TyrA23 or in the *chc2-1* mutant. These results suggested that in addition to CME, another type of endocytosis also participates in the regulation of endocytosis upon hyperosmotic stress.

### 2.3. Membrane Microdomain-Associated Endocytosis Shows an Increase under Hyperosmotic Conditions

**Figure 3:**
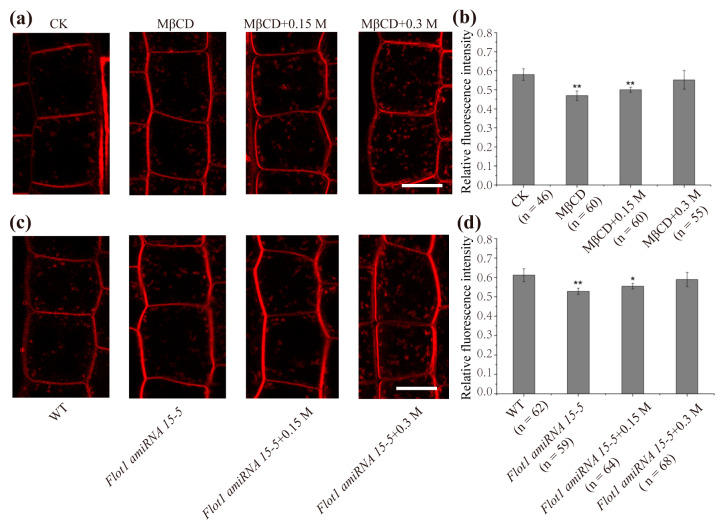
Membrane microdomain-associated endocytosis was involved in increased endocytosis in response to hyperosmotic stress. (**a**) FM4-64 uptake in root epidermal cells of *Arabidopsis thaliana* treated with 1/2 MS (CK) in the absence of MβCD and in the presence of 10 mM MβCD under different conditions: MβCD (1/2 MS), hypermostic stress (0.15 M mannitol, 0.3 M mannitol). (**b**) Quantification of the intracellular relative FM4-64 fluorescence intensity. (**c**) FM-64 uptake in *Arabidopsis* root epidermal cells treated with 1/2 MS, and FM4-64 uptake in *Flot1 amiRNA 15-5* under different conditions: 1/2 MS, hyperosmotic stress (0.15 M mannitol, 0.3 M mannitol). Bar = 10 μm. (**d**) Quantification of the intracellular relative FM4-64 fluorescence intensity. Asterisks mark significant differences (Student’s *t*-test, ** *p* < 0.01; * *p* < 0.05); *n* = number of cells analyzed.

Our results suggest another type of endocytosis involved in the response to hyperosmotic stress in addition to CME. In *Arabidopsis thaliana*, membrane microdomain-associated endocytosis represents a clathrin-independent mechanism. Hence, we assessed whether membrane microdomain-associated endocytosis was involved in adaptation to hyperosmotic stress. Here, we used methyl-β-cyclodextrin (MβCD), which can destroy the membrane raft, to treat WT *Arabidopsis* root cells for 30 min. Notably, there was a 23.2 ± 4.7% reduction in internal FM4-64 signal intensity, indicating that the membrane microdomain-associated endocytosis pathway was inhibited (Figure 3a). Later, we found a 6.4 ± 1.5% and 17.1 ± 4.3% increase in endocytosis, respectively, upon 0.15 M and 0.3 M mannitol treatment in the presence of MβCD compared with single MβCD treatment (Figure 3b). We then observed changes in endocytosis in the artificial small RNA interference transgenic line of Flot1 (*Flot1 amiRNA 15-5*) under hyperosmotic stress. Compared with the control (Figure 3c), the endocytosis of *Flot1 amiRNA 15-5* plants was inhibited to a greater extent (the intracellular relative fluorescence intensity of FM4-64 from 0.61 ± 0.04 to 0.52 ± 0.01) (Figure 3d). After treatment with different concentrations of mannitol, we noted that endocytosis increased by 5.7 ± 1.1% and 13.4 ± 4.3% under 0.15 M and 0.3 M mannitol treatment. (Figure 3d). The previous results proved that endocytosis increased by 18.6 ± 7.5% and 40.3 ± 6.9% under 0.15 M and 0.3 M mannitol treatment, respectively (Figure 2c). However, when inhibitor MβCD or *Flot1 amiRNA 15-5* mutant were used, we found that the increased proportion of endocytosis under hyperosmotic stress significantly decreased (Figure 3b,d). Taken together, these results indicated that in addition to CME, membrane microdomain-associated endocytosis was involved in responding to hyperosmotic stress and was enhanced during this process.

### 2.4. Hyperosmotic Treatments Resulted in a Change in Membrane Raft Microdomains

**Figure 4:**
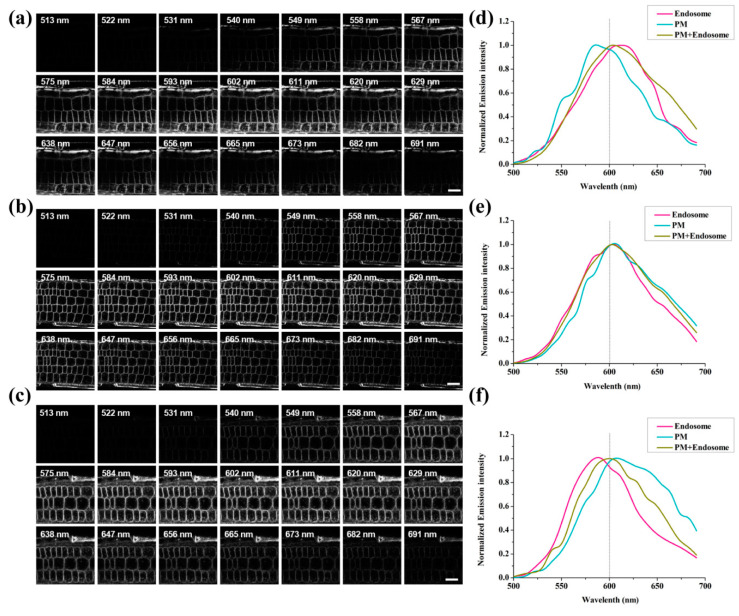
Images series of different spectra taken by the λ-mode using Confocol Laser Scanning Microscope (CLSM) in epidermal cells of *Arabidopsis thaliana* and spectral analysis of root epidermal cells after hyperosmotic treatment. (**a**–**c**). Raw images taken in λ-mode using CLSM in root epidermal cells of *Arabidopsis thaliana* under CK (1/2 MS) (a), and hyperosmotic stress (0.15 M mannitol (b), 0.3 M mannitol (c)). Bar = 10 μm. (**d**–**f**). The emission profiles of di-4-ANEPPDHQ in root epidermal cells of *Arabidopsis thaliana* under different conditions: CK (1/2 MS) (d), hyperosmotic stress (0.15 M mannitol (e), 0.3 M mannitol (f)).

To determine how membrane raft microdomains changed under hyperosmotic stress, we used the membrane microdomain-specific probe di-4-ANEPPDHQ and the analysis method reported previously [29,30]. The emission profiles of di-4-ANEPPDHQ-labeled *Arabidopsis* root epidermal cells excited at a wavelength of 488 nm were recorded using Confocol Laser Scanning Microscope (CLSM) in λ mode (Figure 4a–c). We can judge the change of lipid membrane order by observing the alteration of emission profiles. Among which, the blue-shift indicates more orderly lipid phase while the red-shift is the opposite. As shown in Figure 4, the peak value of endosome exhibited an obvious blue-shift under hyperosmotic stress (Figure 4d–f), suggesting an increased order of endosomes. However, the peak value of the cell membrane displayed a red-shift after mannitol treatment, indicating a decreased order of the cell membrane.

**Figure 5:**
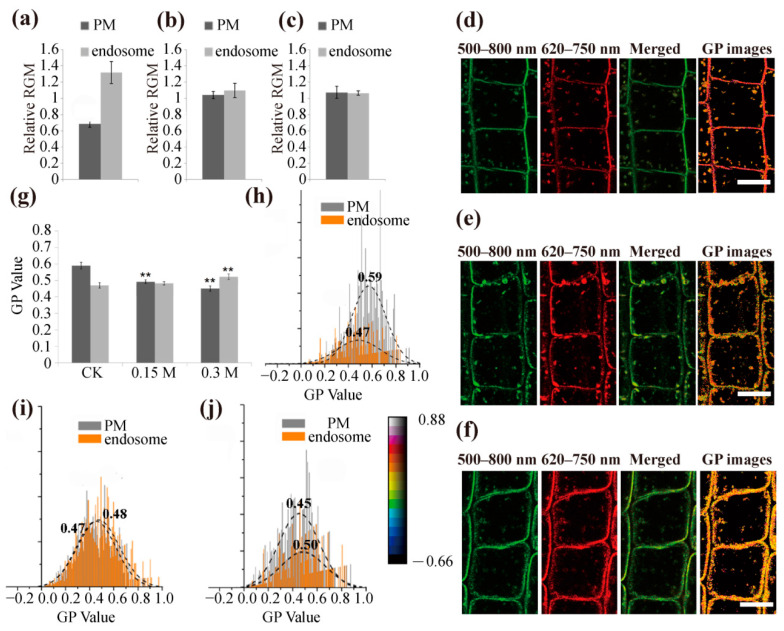
Effect of hyperosmotic stress on membrane order and quantitative imaging of membrane microdomains by generalized polarization processing in *Arabidopsis* epidermal cells. (**a**–**c**) Root epidermal cells in *Arabidopsis thaliana* under different conditions: CK (1/2 MS), hyperosmotic stress (0.15 M mannitol, 0.3 M mannitol), and membrane order was then subsequently quantified using the red-to-green membrane fluorescence ratio (RGM, = I_660_/I_550_). (**d**–**f**) Root epidermal cells in *Arabidopsis thaliana* treated with 1/2 MS (d), 0.15 M mannitol (e), and 0.3 M mannitol (f). CLSM images of the di-4-ANEPPDHQE-labeled cells from the green channel (500–580 nm), red channel (620–750 nm), merged image from both channels, and the generalized polarization (GP) values of each pixel. (**g**) GP values of epidermal cells under different conditions. (**h**–**j**) The distribution of GP values presented in image (d–f). The dashed curves show fitted Gaussian curves from the relative distribution of curvature values. The orange columns are for values measured from endosome structures, and gray columns are for PM regions. Adjusted determination (adjusted R2) was recorded as PM = 0.971 and endosome = 0.917. Asterisks mark significant differences (Student’s *t*-test, ** *p* < 0.01). Bar = 10 μm.

To further discuss and confirm the involvement of membrane microdomains in hyperosmotic stress and determine the changes in membrane order, we measured the fluorescence intensities of the PM and endosome and evaluated the membrane order using the RGM parameter (red-to-green ratio of the membrane or the endosome, RGM = I_660_/I_550_). As shown in Figure 5, the RGM values of PM and endosomes in cells labeled with di-4-ANEPPDHQ under normal conditions were 0.686 ± 0.0013 and 1.317 ± 0.095, respectively. Under hyperosmotic stress, the RGM of PM increased significantly to 1.041 ± 0.016 (0.15 M mannitol) and 1.073 ± 0.021 (0.3 M mannitol), while the RGM of endosomes decreased to 1.098 ± 0.083 (0.15 M mannitol) and 1.065 ± 0.012 (0.3 M mannitol) (Figure 5a–c). Since an increase in membrane order resulted in a reduction in RGM, the membrane order in PM decreased, but the membrane order in endosomes increased, implying internalization of membrane microdomains in response to hyperosmotic stress.

We next performed a GP (generalized polarization) analysis on the order of membrane lipids. Previously, an increased order of membrane lipids was found to result in high GP values [30]. We obtained the GP diagram following the method of Zhao et al. [30] and then observed the distribution of GP values of root epidermal cells in *Arabidopsis thaliana* (Figure 5d–f) under different hyperosmotic conditions. The minimum GP value was set to 0.66 (dark), and the maximum value was set to 0.88 (silver). The PM has a higher order (red) than the endosome (yellow) under normal circumstances (Figure 5d). Under hyperosmotic treatment, the PM showed a decreased order (yellow) compared with the control (red). (Figure 5e–f). Then, we counted the recorded pixels in each GP value under different conditions and made the corresponding histograms (Figure 5g–j). We performed a single-peak fitting analysis of the histogram of the GP values from the PM and endosome regions and noted that the GP peak values of PM and endosomes were 0.59 ± 0.02 and 0.47 ± 0.02, respectively, in the control group (Figure 5h). After hyperosmotic treatment, the value of PM decreased to 0.48 ± 0.01 (0.15 M mannitol) and 0.45 ± 0.01 (0.3 M mannitol). In contrast, the endosome values were 0.47 ± 0.01 and 0.50 ± 0.01, respectively (Figure 5i,j), displaying an increased order compared with the control. These results suggested that the circulation of the cell membrane structure of the root epidermal cells was promoted upon hyperosmotic treatment, that the order of PM of the epidermal cells was reduced, and that the order of endosomes was increased under hyperosmotic treatment.

## 3. Discussion

In nature, plant cells are inevitably affected by various abiotic stresses in their lives, such as osmotic stress, drought stress, and heat stress [31,32]. To adapt to the stress caused by changes in the external environment, plants need to respond to these stresses through various biochemical processes [33]. Hyperosmotic stress, as one of the most common stresses in plants, seriously affects the growth and development of plants [18,34,35]. Additionally, since hyperosmotic stress directly causes cell dehydration and plasma-wall separation, the restoration of cell turgor pressure and plasma membrane remodeling are essential for maintaining plant cell life, and endocytosis is a key factor for this process [4].

Here, we first focused on the most important endocytosis pathway in plant cells and investigated the response of clathrin-mediated endocytosis to osmotic stress. Under confocal microscopy, hyperosmotic stress promoted the internalization of CLC-GFP, which is consistent with previous research [21]. Using VA-TIRFM technology, we analyzed the dynamics of CLC-GFP at the plasma membrane and found that CLC-GFP formed fluorescent spots within the diffraction limit on the cell membrane. The histogram of diffusion coefficients of CLC-GFP showed a single-peak distribution under normal conditions and displayed a broad, bimodal distribution upon hyperosmotic stress, suggesting the existence of two subpopulations of diffusing CLC-GFP molecules (Figure 1f). Of these subpopulations, one subpopulation possessed a diffusion coefficient that is approximately 125% of the diffusion coefficient observed under normal conditions. Another subpopulation of CLC-GFP diffused slowly with Ĝ = 3.83 ± 0.12 × 10 ^−^^4^ μm^2^/s, which was 5 times lower than the control. These results indicated that CLC-GFP tended to have higher diffusion coefficients and that a fraction of CLC-GFP molecules underwent slower diffusion upon hyperosmotic stress. Furthermore, we found an increased motion range of CLC-GFP compared with the control. Meanwhile, the fluorescence intensity of CLC-GFP on the plasma membrane decreased, indicating reduced CLC-GFP molecules on the PM (Figure 1c). The CME process occurs through a sequence of regulated events, including the assembly and maturation of clathrin coat pits [36]. Since the assembly of clathrin molecules results in the aggregation of CLC-GFP, the reduction of the fluorescence intensity at the PM and the decreased diffusion coefficients of a fraction of CLC-GFP, as well as the increased motion range, may represent the formation of clathrin-coated particles and the internalization of CLC-GFP. We further explored endocytosis in root epidermal cells in response to hyperosmotic stress and found that endocytosis increased by 18.6% (0.15 M mannitol) and 40.3% (0.3 M mannitol). The average endocytosis increase slowed down after the inhibition of the CME using TyrA23, indicating that CME contributed to the enhanced endocytosis under hyperosmotic conditions (Figure 2). Interestingly, we found that there was still a small increase in endocytosis when CME was inhibited; hence, another endocytosis pathway responded to hyperosmotic stress. Similar results were obtained using the clathrin loss-of-function mutant *chc2*-*1*.

In addition to CME, an additional endocytic pathway-microdomain-associated endocytosis was revealed in *Arabidopsis thaliana* [11]. In the present study, we also found that the increase in endocytosis slowed down when microdomain-associated endocytosis was restrained or in *Flot1 amiRNA* plants, demonstrating that endocytosis mediated by membrane microdomains was enhanced under hyperosmotic conditions (Figure 3). In a previous study, the plant cytoplasmic membrane aquaporin PIP2;1 was endocytosed mainly through clathrin-dependent pathway under normal conditions. When plant cells were subjected to NaCl treatment, the endocytosis of PIP2;1 was intensified, and membrane microdomain-associated endocytosis was activated, which cooperated with the endocytosis of PIP2;1 under NaCl treatment [10]. Hyperosmotic conditions induce a decrease in membrane tension and cell shrinkage, resulting in an excess of PM needing to be internalized for adaptation to stress [37]. Here, our results demonstrated that clathrin-mediated and membrane microdomain-associated endocytosis cooperated with cellular adaptation to hyperosmotic stress in *Arabidopsis thaliana*. Since CME and microdomain-associated endocytosis selectively mediate the uptake of specific PM proteins [5,38], the endocytosis of specific PM proteins under hyperosmotic stress needs to be further illustrated.

To further explore the involvement of membrane microdomain-associated endocytosis under hyperosmotic stress, we used the new type of phenethyl dye Di-4-ANEPPDHQ. As a recognized low-toxicity fluorescent probe, Di-4-ANEPPDHQ has been well used in animal and plant cells [29,30,39,40,41,42,43]. The emission peak values of this probe display a red-shift from the lipid-ordered phase to the lipid-disordered phase [39,44]. In our results, the peak value of the cell membrane displayed a red-shift after mannitol treatment (Figure 4d–f), indicating a decreased order of the cell membrane and internalization of plasma membrane microdomains. By comparing the RGM of PM and endosomes in epidermal cells before and after hyperosmotic stress, we found that the RGM value of PM increased while the RGM of endosomes decreased upon hyperosmotic treatment (Figure 5a–c). We carried out normalized numerical GP analysis and found that the GP peak value of PM decreased while the GP peak value of endosomes increased (Figure 5g–j). According to a previous report, conditions inducing an increase in PM order resulted in a significantly lower RGM [42]. Moreover, membrane microdomains can be defined by their high orders and can be visualized through membrane areas with high GP values [45,46]. Here, our results suggested that hyperosmotic stress induces fewer ordered domains in the PM but more ordered domains in endosomes, further supporting the involvement of microdomain-associated endocytosis in adaptation to hyperosmotic stress.

Our results support that both CME and membrane microdomain-associated endocytosis contribute to cell adaptation to hyperosmotic stress. Membrane rafts are well known to be dynamic membrane microdomains rich in sterols and sphingolipids with a size of nanometers. The membrane raft provides a platform for the parking of proteins and is an indispensable platform for membrane-based signal transduction and protein sorting [47,48]. Here, our study showed that a large number of ordered microdomains in *Arabidopsis* root epidermal cells were internalized, which were most likely to promote the movement of many signaling proteins harboring these microdomains. The detailed process of the regulation of these signaling molecules in the microdomain-associated endocytosis pathway under hyperosmotic stress is worth researching.

## 4. Materials and Methods

### 4.1. Plant Material and Growth Conditions

Seeds of wild-type *Arabidopsis thaliana* (Col-0), CLC-GFP [11,26], and the *Arabidopsis* mutant *chc 2*-*1* [49] and *Flot1 amiRNA15-5* [11] were stratified for 2 days in the dark at 4 °C. Seedlings were germinated and grown vertically on 1/2 MS solidified with 0.8% agar at a pH of 5.8 at 22 °C under a 16/8-h light–dark cycle.

### 4.2. Drug Treatments and FM4-64 Staining and Imaging

Four-day-old seedlings were incubated in 1/2 MS medium (CK), 1/2 MS medium supplemented with mannitol (0.15 M and 0.3 M final concentration) together with 3 μM FM4-64 (Merck KGaA, Darmstadt, Germany) for 5 min at room temperature and then observed under a Leica SP5 confocal microscope (Leica Microsystems GmbH, Wetzlar, Germany).

For the inhibitor treatments, 4-day-old seedlings were incubated in MβCD (Merck KGaA, Darmstadt, Germany) (diluted to 10 mM with 1/2 MS medium) or TyrA23 (Merck KGaA, Darmstadt, Germany) (diluted to 50 μM with 1/2 MS medium) for 30 min, followed by incubation for an additional 5 min by the addition of 3 μM FM4-64 (final concentration) in the absence or presence of mannitol (0.15 M or 0.3 M final concentration). After rinsing with the same solution (without FM4-64) for 1 min, the seedlings were observed under a Leica SP5 confocal microscope (Leica Microsystems GmbH, Wetzlar, Germany) fitted with a 63× oil objective, excited using 488 nm wavelength for GFP and 561 nm wavelength for FM4-64 respectively.

### 4.3. Quantitative Analysis of Endocytosis

For evaluation of changes in endocytosis, the average number of punctate structures labeled with FM4-64 in a single cell under normal conditions or mannitol stress was quantified using ImageJ (Image high-energy version 1.4.3.67, National Institutes of Mental Health, Bethesda, MD, USA). Meanwhile, the mean fluorescence intensity of punctate structures inside the cell stained with FM4-64, excluding the PM, was also measured with ImageJ software according to Zwiewk et al. [21]. The mean fluorescence intensity of internalized CLC-GFP colocalized with FM4-64 was also measured using ImageJ. Approximately 20 cells from at least three different seedlings were used for analysis.

### 4.4. Di-4-ANEPPDHQ Staining and Imaging

Four-day-old seedlings were placed in a small tube and incubated with di-4-ANEPPDHQ (Invitrogen, Shanghai, China, stock solution in DMSO, diluted to a final concentration of 3 μM with 1/2 MS medium) for 5 min at room temperature without light in the absence or presence of mannitol (0.15 M or 0.3 M final concentration), and the images were captured with a Zeiss LSM 780 confocal microscope (Carl Zeiss, Oberkochen, Baden-Württemberg, Germany) fitted with a 63× oil-immersion objective lens. After excitation at 488 nm, the images were scanned from 495 to 691 nm at 9 nm intervals in x-y-lambda mode. To draw the emission spectra in root epidermal cells, 20 rectangular regions of interest (ROIs) within the plasma membrane and endosomes of the same size were randomly selected, and the fluorescence intensity value of the selected ROI area was measured by the Multi Measure plug-in at each wavelength. Three independent biological replicates were taken for each region and treatment. Mean values were used for the counter emission spectra.

For RGM (red-to-green ratio of membrane fluorescence) analysis, di-4-ANEPPDHQ-stained samples were excited at 488 nm, and emission intensities were acquired between 500–580 nm (green image) and between 620–750 nm (red image). Radiometric imaging was performed using ImageJ software.

### 4.5. Membrane Order Determination by GP Processing

After acquisition images of root epidermal cells in dual channels, 500–580 nm for the lipid-ordered phase and 620–750 nm for the lipid-disordered phase, a new image representing the membrane lipid order was obtained by calculating the GP value using ImageJ and the macro provided by Owen et al. [45]. GP images were generated according to the following calculation: GP = (I_500-580_ − G × I_620-750_)/(I_500-580_ + G × I_620-750_) as described previously [29,43]. In addition, the recorded pixels in each GP value in different regions were quantified, and histograms were generated. The peak GP values from endosomes and the PM were calculated using the Gaussian-fitted curves on these histograms.

### 4.6. VA-TIRFM and Fluorescence Image Analysis

Four-day-old CLC-GFP transgenic plants were mounted on glass slides and observed under a variable-angle total internal reflection fluorescence microscope (VA-TIRFM). CLC-GFP were excited with 488 nm laser lines. The images were collected according to Lv et al. [50], and the trajectory of the CLC-GFP protein was tracked using ImageJ software. The kinetic parameters were analyzed with MATLAB R2009b (Mathworks, Natick, Massachusetts, USA) and Origin 2019. The diffusion coefficient and motion range were analyzed as described previously [10,11,50].

### 4.7. Data Analysis

Significant differences among experimental data are denoted by asterisks (* *p* < 0.05, ** *p* < 0.01; Student’s *t* test). The error bars represent the standard deviation (SD) of three independent biological replicates. Graphs were created using software Prism 7 (GraphPadSoftware, San Diego, State of California, USA) and Origin 2019 (OriginLab Corporation, Northampton, Massachusetts, USA).

## Data Availability

The data presented in this study are available in article.

## References

[1] Wolfe J., Steponkus P.L. (1983). Mechanical properties of the plasma membrane of isolated plant protoplasts: Mechanism of hyperosmotic and extracellular freezing injury. Plant Physiol..

[2] Tran L.S., Nakashima K., Shinozaki K., Yamaguchi-Shinozaki K. (2007). Plant gene networks in osmotic stress response: From genes to regulatory networks. Methods Enzymol..

[3] Rodas-Junco B.A., Racagni-Di-Palma G.E., Canul-Chan M., Usorach J., Hernández-Sotomayor S.M.T. (2021). Link between lipid second messengers and osmotic stress in plants. Int. J. Mol. Sci..

[4] López-Hernández T., Haucke V., Maritzen T. (2020). Endocytosis in the adaptation to cellular stress. Cell Stress.

[5] Fan L., Li R., Pan J., Ding Z., Lin J. (2015). Endocytosis and its regulation in plants. Trends Plant Sci..

[6] Murphy A.S., Bandyopadhyay A., Holstein S.E., Peer W.A. (2005). Endocytotic cycling of PM proteins. Annu. Rev. Plant Biol..

[7] Bandmann V., Homann U. (2012). Clathrin-independent endocytosis contributes to uptake of glucose into BY-2 protoplasts. Plant J..

[8] Bitsikas V., Correa I.R., Nichols B.J. (2014). Clathrin-independent pathways do not contribute significantly to endocytic flux. Elife.

[9] Kaksonen M., Roux A. (2018). Mechanisms of clathrin-mediated endocytosis. Nat. Rev. Mol. Cell Biol..

[10] Li X., Wang X., Yang Y., Li R., He Q., Fang X., Luu D.T., Maurel C., Lin J. (2011). Single-molecule analysis of PIP2; 1 dynamics and partitioning reveals multiple modes of *Arabidopsis* plasma membrane aquaporin regulation. Plant Cell.

[11] Li R., Liu P., Wan Y., Chen T., Wang Q., Mettbach U., Baluska F., Samaj J., Fang X., Lucas W.J. (2012). A membrane microdomain-associated protein, *Arabidopsis* Flot1, is involved in a clathrin-independent endocytic pathway and is required for seedling development. Plant Cell.

[12] Cao Y., He Q., Qi Z., Zhang Y., Lu L., Xue J., Li J., Li R. (2020). Dynamics and Endocytosis of Flot1 in *Arabidopsis* Require CPI1 Function. Int. J. Mol. Sci..

[13] Zhang L., Xing J., Lin J. (2019). At the intersection of exocytosis and endocytosis in plants. New Phytol..

[14] Munns R., Tester M. (2008). Mechanisms of salinity tolerance. Annu. Rev. Plant Biol..

[15] Ismail A., Takeda S., Nick P. (2014). Life and death under salt stress: Same players, different timing?. J. Exp. Bot..

[16] Zhu J.K. (2016). Abiotic stress signaling and responses in plants. Cell.

[17] Yang Y., Yan G. (2018). Unraveling salt stress signaling in plants. J. Integr. Plant Biol..

[18] Chen K., Song M., Guo Y., Liu L., Xue H., Dai H., Zhang Z. (2019). MdMYB46 could enhance salt and osmotic stress tolerance in apple by directly activating stress-responsive signals. Plant Biotechnol. J..

[19] Kuraoda R., Kato M., Tsuge T., Aoyama T. (2021). *Arabidopsis* phosphatidylinositol 4-phosphate 5-kinase genes *PIP5K7*, *PIP5K8*, and *PIP5K9* are redundantly involved in root growth adaptation to osmotic stress. Plant J..

[20] Dai J., Sheetz M.P. (1995). Regulation of endocytosis, exocytosis, and shape by membrane tension. Cold Spring Harb. Symp. Quant. Biol..

[21] Zwiewka M., Nodzynski T., Robert S., Vanneste S., Friml J. (2015). Osmotic stress modulates the balance between exocytosis and clathrin-mediated endocytosis in *Arabidopsis thaliana*. Mol. Plant..

[22] Chadwick S.R., Wu J.Z., Freeman S.A. (2021). Solute transport controls membrane tension and organellar volume. Cell. Physiol. Biochem..

[23] Ferguson J.P., Huber S.D., Willy N.M., Aygün E., Goker S., Atabey T., Kural C. (2017). Mechanoregulation of clathrin-mediated endocytosis. J. Cell Sci..

[24] Bucher D., Frey F., Sochacki K.A., Kummer S., Bergeest J.P., Godinez W.J., Kräusslich H.G., Rohr K., Taraska J.W., Schwarz U.S. (2018). Clathrin-adaptor ratio and membrane tension regulate the flat-to-curved transition of the clathrin coat during endocytosis. Nat. Commun..

[25] Mazheika I., Voronko O., Kamzolkina O. (2020). Early endocytosis as a key to understanding mechanisms of plasma membrane tension regulation in filamentous fungi. Biol. Cell..

[26] Konopka C.A., Backues S.K., Bednarek S.Y. (2008). Dynamics of *Arabidopsis* dynamin-related protein 1C and a clathrin light chain at the plasma membrane. Plant Cell..

[27] Ito E., Fujimoto M., Ebine K., Uemura T., Ueda T., Nakano A. (2012). Dynamic behavior of clathrin in *Arabidopsis thaliana* unveiled by live imaging. Plant J..

[28] Nagy E. (2012). 2-Molecular Diffusion.

[29] Zhao X., Li R., Lu C., Baluska F., Wan Y. (2015). Di-4-ANEPPDHQ, a fluorescent probe for the visualisation of membrane microdomains in living *Arabidopsis thaliana* cells. Plant Physiol. Biochem..

[30] Zhao X., Zhang X., Qu Y., Li R., Baluška F., Wan Y. (2015). Mapping of membrane lipid order in root apex zones of *Arabidopsis thaliana*. Front. Plant Sci..

[31] Sun X., Wang Y., Sui N. (2018). Transcriptional regulation of bHLH during plant response to stress. Biochem. Biophys. Res. Commun..

[32] Gong Z., Xiong L., Shi H., Yang S., Herrera-Estrella L.R., Xu G., Chao D.Y., Li J., Wang P.Y., Qin F. (2020). Plant abiotic stress response and nutrient use efficiency. Sci. China Life Sci..

[33] Peck S., Mittler R. (2020). Plant signaling in biotic and abiotic stress. J. Exp. Bot..

[34] Ueda M., Tsutsumi N., Fujimoto M. (2016). Salt stress induces internalization of plasma membrane aquaporin into the vacuole in *Arabidopsis thaliana*. Biochem. Biophys. Res. Commun..

[35] Hong Y., Yuan S., Sun L., Wang X., Hong Y. (2018). Cytidinediphosphate-diacylglycerol synthase 5 is required for phospholipid homeostasis and is negatively involved in hyperosmotic stress tolerance. Plant J..

[36] Mettlen M., Chen P.H., Srinivasan S., Danuser G., Schmid S.L. (2018). Regulation of clathrin-mediated endocytosis. Annu. Rev. Biochem..

[37] Kozera L., White E., Calaghan S. (2009). Caveolae act as membrane reserves which limit mechanosensitive I(Cl,swell) channel activation during swelling in the rat ventricular myocyte. PLoS ONE.

[38] Wang S., Singh R.D., Godin L., Pagano R.E., Hubmayr R.D. (2011). Endocytic response of type I alveolar epithelial cells to hypertonic stress. Am. J. Physiol. Lung. Cell Mol. Physiol..

[39] Owen D.M., Lanigan P.M., Dunsby C., Munro I., Grant D., Neil M.A., French P.M., Magee A.I. (2006). Fluorescence lifetime imaging provides enhanced contrast when imaging the phase-sensitive dye di-4-ANEPPDHQ in model membranes and live cells. Biophys. J..

[40] Obaid A.L., Loew L.M., Wuskell J.P., Salzberg B.M. (2004). Novel naphthylstyryl-pyridinium potentiometric dyes offer advantages for neural network analysis. J. Neurosci. Methods.

[41] Owen D.M., Gaus K. (2010). Optimized time-gated generalized polarization imaging of Laurdan and di-4-ANEPPDHQ for membrane order image contrast enhancement. Microsc. Res. Tech..

[42] Grosjean K., Der C., Robert F., Thomas D., Mongrand S., Simon-Plas F., Gerbeau-Pissot P. (2018). Interactions between lipids and proteins are critical for organization of plasma membrane-ordered domains in tobacco BY-2 cells. J. Exp. Bot..

[43] Imran M., Sergent O., Tête A., Gallais I., Chevanne M., Lagadic-Gossmann D., Podechard N. (2018). Membrane remodeling as a key player of the hepatotoxicity induced by co-exposure to benzo[a]pyrene and ethanol of obese zebrafish larvae. Biomolecules.

[44] Jin L., Millard A.C., Wuskell J.P., Dong X., Wu D., Clark H.A., Loew L.M. (2006). Characterization and application of a new optical probe for membrane lipid domains. Biophys. J..

[45] Owen D.M., Rentero C., Magenau A., Abu-Siniyeh A., Gaus K. (2012). Quantitative imaging of membrane lipid order in cells and organisms. Nat. Protoc..

[46] Aron M., Browning R., Carugo D., Sezgin E., Bernardino de la Serna J., Eggeling C., Stride E. (2017). Spectral imaging toolbox: Segmentation, hyperstack reconstruction, and batch processing of spectral images for the determination of cell and model membrane lipid order. BMC Bioinform..

[47] Pike L.J. (2004). Lipid rafts: Heterogeneity on the high seas. J. Biochem..

[48] Pike L.J. (2005). Growth factor receptors, lipid rafts and caveolae: An evolving story. Biochim. Biophys. Acta..

[49] Zhang X., Cui Y., Yu M., Su B., Gong W., Baluška F., Komis G., Šamaj J., Shan X., Lin J. (2019). Phosphorylation-mediated dynamics of nitrate transceptor NRT1.1 regulate auxin flux and nitrate signaling in lateral root growth. Plant Physiol..

[50] Lv X., Jing Y., Xiao J., Zhang Y., Zhu Y., Julian R., Lin J. (2017). Membrane microdomains and the cytoskeleton constrain AtHIR1 dynamics and facilitate the formation of an AtHIR1-associated immune complex. Plant J..

